# Home blood pressure-lowering effect of esaxerenone versus trichlormethiazide for uncontrolled hypertension: a predefined subanalysis of the EXCITE-HT randomized controlled trial by basal calcium channel blocker versus angiotensin receptor blocker

**DOI:** 10.1038/s41440-024-01887-1

**Published:** 2024-10-12

**Authors:** Kazuomi Kario, Hiroyuki Ohbayashi, Masami Hashimoto, Naoki Itabashi, Mitsutoshi Kato, Kazuaki Uchiyama, Kunio Hirano, Noriko Nakamura, Takahide Miyamoto, Hirotaka Nagashima, Hidenori Ishida, Yusuke Ebe, Tsuguru Hatta, Toshiki Fukui, Tatsuo Shimosawa, Tomohiro Katsuya, Takashi Taguchi, Ayumi Tanabe, Mitsuru Ohishi, on behalf of the EXCITE-HT investigators

**Affiliations:** 1 Division of Cardiovascular Medicine, Department of Medicine, Jichi Medical University School of Medicine, Shimotsuke, Tochigi Japan; 2 Tohno Chuo Clinic, Mizunami, Gifu Japan; 3 Hashimoto Kidney Clinic, Fukuyama, Hiroshima Japan; 4 Itabashi Diabetes and Dermatology Medical Clinic, Koga, Ibaraki Japan; 5 Kato Clinic of Internal Medicine, Katsushika-ku, Tokyo Japan; 6 Uchiyama Clinic, Joetsu, Niigata Japan; 7 Hirano Clinic, Morioka, Iwate Japan; 8 Primula Clinic, Kagoshima, Kagoshima Japan; 9 Miyamoto Clinic of Internal Medicine, Matsumoto, Nagano Japan; 10 Tokyo Center Clinic, Chuo-ku, Tokyo Japan; 11 Akaicho Clinic, Chiba, Chiba Japan; 12 Ebe Clinic, Nagaoka, Niigata Japan; 13 Hatta Medical Clinic, Kyoto, Kyoto Japan; 14 Olive Takamatsu Medical Clinic, Takamatsu, Kagawa Japan; 15 Department of Clinical Laboratory, School of Medicine, International University of Health and Welfare, Narita, Chiba Japan; 16 Katsuya Clinic, Amagasaki, Hyogo Japan; 17 Primary Medical Science Department, Medical Affairs Division, Daiichi Sankyo Co. Ltd., Chuo-ku, Tokyo Japan; 18 Data Intelligence Department, Daiichi Sankyo Co. Ltd., Shinagawa-ku, Tokyo Japan; 19 Department of Cardiovascular Medicine and Hypertension, Graduate School of Medical and Dental Sciences, Kagoshima University, Kagoshima, Kagoshima Japan

**Keywords:** Angiotensin II receptor blockers, Calcium channel blockers, Esaxerenone, Essential hypertension, Trichlormethiazide

## Abstract

This prespecified subanalysis of the multicenter, randomized, open-label, parallel-group EXCITE-HT study aimed to examine the non-inferiority of esaxerenone to trichlormethiazide as a second-line antihypertensive agent according to the basal antihypertensive agent used (angiotensin receptor blocker [ARB] or calcium channel blocker [CCB]). The primary endpoint, change in morning home systolic/diastolic blood pressure (SBP/DBP) from baseline to end of treatment was similar between the two groups (intergroup difference in least squares mean change [95% confidence interval]: −1.3 [−3.8, 1.3]/−0.2 [−1.6, 1.3] mmHg for ARB; −2.7 [−4.2, −1.2]/−0.8 [−1.7, 0.1] mmHg for CCB). The respective incidences of serum potassium levels <3.5 mEq/L and ≥5.5 mEq/L in the ARB subgroup were 3.4% and 4.2% for esaxerenone and 7.9% and 0% for trichlormethiazide; in the CCB subgroup, they were 2.8% and 0.6% for esaxerenone and 13.9% and 1.2% for trichlormethiazide, respectively. The incidence of uric acid level ≥7.0 mg/dL was numerically higher in the trichlormethiazide group than the esaxerenone group in both the ARB and CCB subgroups. The non-inferiority of esaxerenone to trichlormethiazide in lowering morning home BP was demonstrated regardless of whether the basal antihypertensive agent was an ARB or CCB. Esaxerenone with a CCB showed superiority to trichlormethiazide in lowering SBP, without any new safety concerns. Serum potassium levels tended to be higher when esaxerenone was combined with an ARB than with a CCB, but this can be mitigated if administered according to the package insert.

**Figure:**
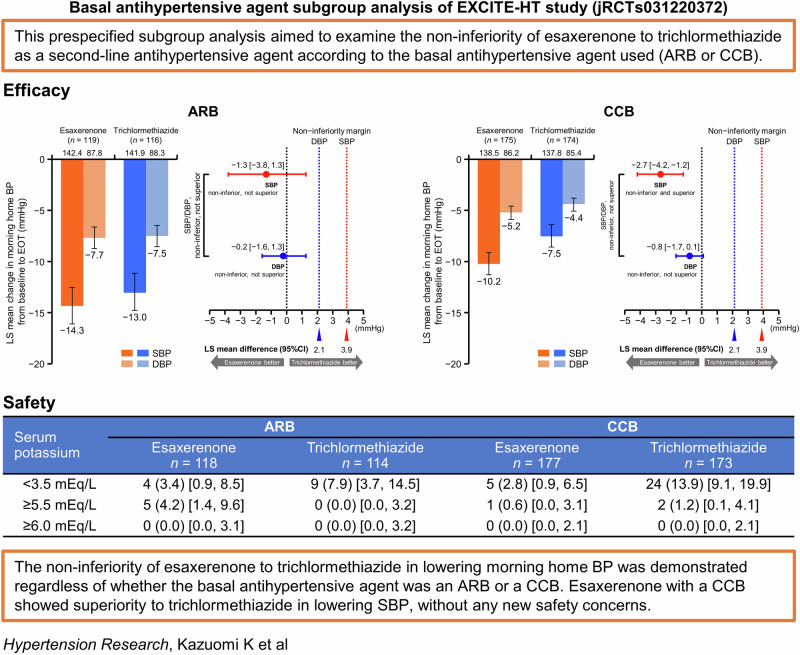
A subgroup analysis of the EXCITE-HT study according to basal antihypertensive agent demonstrated the non-inferiority of esaxerenone to trichlormethiazide in lowering morning home BP regardless irrespective of the basal antihypertensive agent. Esaxerenone with a CCB showed superiority to trichlormethiazide in lowering SBP, without any new safety concerns.

A subgroup analysis of the EXCITE-HT study according to basal antihypertensive agent demonstrated the non-inferiority of esaxerenone to trichlormethiazide in lowering morning home BP regardless irrespective of the basal antihypertensive agent. Esaxerenone with a CCB showed superiority to trichlormethiazide in lowering SBP, without any new safety concerns.

## Abstract (web summary)

A subgroup analysis of the EXCITE-HT study according to basal antihypertensive agent demonstrated the non-inferiority of esaxerenone to trichlormethiazide in lowering morning home BP regardless irrespective of the basal antihypertensive agent. Esaxerenone with a CCB showed superiority to trichlormethiazide in lowering SBP, without any new safety concerns.

## Introduction

Adequate blood pressure (BP) control is required for the prevention of cardiovascular and cerebrovascular events [1, 2]. Despite the availability of numerous antihypertensive agents in clinical use, adequate BP management remains elusive for some patients [3, 4], particularly in Japan. This phenomenon, known as the ‘hypertension paradox’, refers to the increasing population of patients with uncontrolled BP, despite advancements in therapeutic interventions [5–7]. One potential explanation for this paradox is the insufficient BP-lowering effect of single antihypertensive agents, highlighting the importance of combination therapy in achieving optimal BP management.

According to the Japanese Society of Hypertension (JSH) 2019 guideline, the primary antihypertensive agents currently used as first-line treatment are calcium channel blockers (CCBs), renin–angiotensin system (RAS) inhibitors (angiotensin II receptor blockers [ARBs] and angiotensin-converting enzyme inhibitors), and diuretics (thiazide diuretics and loop diuretics) in hypertensive patients without contraindication or positive indication [8]. In real-world clinical practice in Japan, ARBs and CCBs are more commonly prescribed than other antihypertensive agents, with prescription rates of 40%–63% for ARBs and 68–74% for CCBs [9, 10].

ARBs are expected to provide organ-protective effects in addition to their antihypertensive effects [11], but they are also associated with adverse effects, including aldosterone breakthrough induction when taken long-term, and serum potassium elevation [12, 13]. CCBs are characterized by their low metabolic impact, reliable antihypertensive effects regardless of patient demographics, and ease of use in the elderly [14, 15]. It has been reported that only approximately 34–40% of patients achieve target BP with ARB or CCB monotherapy [16], reemphasizing that a combination of two or more antihypertensive agents is generally recommended for the management of hypertension.

Esaxerenone is a next-generation non-steroidal mineralocorticoid receptor blocker (MRB) that offers higher selectivity and potency, longer half-life, and more favorable bioavailability compared with other MRBs [17, 18]. The phase 3 ESAX-HTN study demonstrated the non-inferiority of esaxerenone (2.5 mg/day) to eplerenone (50 mg/day) in its BP-lowering effect [19].

The EXCITE-HT study was the first randomized controlled trial that demonstrated the non-inferiority of esaxerenone to trichlormethiazide in terms of antihypertensive effects based on morning home BP as a second-line antihypertensive agent alongside another agent class in patients with uncontrolled essential hypertension [20, 21], and no new safety concerns were raised compared with previous esaxerenone clinical studies [22]. Furthermore, the incidences of adverse events such as low serum potassium and hyperuricemia were lower with esaxerenone than with trichlormethiazide. However, the clinical question remains whether there may be differences in the efficacy and safety of esaxerenone combination therapy depending on the basal antihypertensive agent used (ARB or CCB).

This EXCITE-HT study subgroup analysis, prespecified in the statistical analysis plan, aimed to examine the non-inferiority of esaxerenone to trichlormethiazide as a second-line antihypertensive agent according to the type of basal antihypertensive agent used (ARB or CCB).

## Methods

### Study design

Details of the EXCITE-HT study have been published previously [20, 21]. Briefly, the EXCITE-HT study was a multicenter (54 sites), randomized, open-label, parallel-group study conducted between December 2022 and September 2023, designed to determine the non-inferiority of esaxerenone versus trichlormethiazide in its antihypertensive effect [20, 21]. The study protocol of the main study was approved by the Certified Review Board of Hattori Clinic (CRB3180027) and was registered at the Japan Registry of Clinical Trials under the identifier jRCTs031220372 (https://jrct.niph.go.jp/en-latest-detail/jRCTs031220372). All patients provided written informed consent before enrollment. The study was conducted in accordance with the principles of the Declaration of Helsinki and the Clinical Trials Act in Japan.

### Patients

Patients aged ≥20 years who received prior treatment with either one ARB or one CCB at the same dose for ≥4 weeks before registration and had mean morning home systolic BP (SBP) ≥ 125 mmHg and/or diastolic BP (DBP) ≥ 75 mmHg were included. Patients aged ≥75 years, with cerebrovascular disease or proteinuria-negative chronic kidney disease were eligible if they had a mean morning home SBP ≥ 135 mmHg and/or a DBP ≥ 85 mmHg [20, 21].

### Study interventions

Information regarding patient and drug allocation has been reported previously [20]. The starting dose of esaxerenone was 2.5 mg/day, and the maximum dose was 5 mg/day. In patients with an estimated glomerular filtration rate (eGFR) of 30–59 mL/min/1.73 m^2^ or those with diabetes mellitus and albuminuria or proteinuria at baseline, the starting dose was 1.25 mg/day. Esaxerenone was administered for 12 weeks according to the Japanese package insert [23]. The dose could be gradually increased to 5 mg/day based on BP and serum potassium level at either 4 or 8 weeks of esaxerenone treatment.

Trichlormethiazide was administered at the physician’s discretion according to the Japanese package insert and the JSH 2019 guidelines [8, 24], in which the recommended starting dose of trichlormethiazide is ≤1 mg/day. The dose was increased at Weeks 4 and 8 according to the physician’s judgment based on the patient’s condition.

Basal antihypertensive agents (ARB or CCB) were administered at a continuous dose until the end of treatment (EOT). The use of antihypertensive agents other than the assigned medication was prohibited.

### Study measurements

Home BP measurements were taken twice daily throughout the study period, in the morning and at bedtime, using an upper arm cuff sphygmomanometer (HCR-7501T, OMRON Healthcare Co., Ltd., Japan) [20]. The average of two measurements at each timepoint within the last 5 days before the patient’s visit was calculated and recorded. Office BP was also measured twice daily [8] at specific timepoints, and the average of two measurements at each timepoint was calculated and recorded.

### Study endpoints

The primary endpoint was the change in morning home SBP/DBP from baseline to EOT. Secondary endpoints included the change in bedtime home and office SBP/DBP from baseline to EOT and the change in urinary albumin-to-creatinine ratio (UACR) and serum N-terminal pro-brain natriuretic peptide (NT-proBNP) levels from baseline to Week 12. Safety endpoints included the change from baseline in creatinine-based eGFR (eGFR_creat_) and serum potassium; the proportion of patients with a serum potassium level ≤3.5 mEq/L, ≥5.5 mEq/L, and ≥6.0 mEq/L; and the proportion of patients with a uric acid (UA) level ≥7.0 mg/dL.

### Sample size and statistical analyses

A target sample size was set for the main study but not for this subgroup analysis [20, 21]. The data were analyzed by subgroup of basal antihypertensive agent (ARB or CCB). The full analysis set (FAS) was used for the main analysis, and the per protocol set (PPS) was used for the ancillary analysis as in the main study. The statistical methods used were the same as those for the primary analysis [20, 21]. Briefly, the non-inferiority of esaxerenone to trichlormethiazide was considered when the upper limit of the two-sided 95% confidence interval (CI) for the difference in both SBP and DBP change between esaxerenone and trichlormethiazide was <3.9 mmHg and <2.1 mmHg, respectively. The superiority of esaxerenone to trichlormethiazide was considered when the upper limit of the two-sided 95% CI was <0 mmHg. All statistical analyses had a two-sided significance level of 5% (unless otherwise stated) and were conducted using SAS version 9.4 or higher (SAS Institute Inc., Cary, NC, USA).

## Results

### Patients

In the main study, 600 eligible patients were randomly assigned to the esaxerenone and trichlormethiazide groups (*n* = 295 and 290 in the FAS and 275 and 290 in the PPS, respectively). Among these, 119 patients in the esaxerenone group and 116 patients in the trichlormethiazide group were included in the ARB subgroup; 176 and 174 patients in the esaxerenone and trichlormethiazide groups, respectively, were included in the CCB subgroup (FAS).

Baseline patient characteristics are summarized in Table 1. In the ARB subgroup, the mean morning home SBP/DBP was 142.4/87.8 and 141.9/88.3 mmHg in the esaxerenone and trichlormethiazide groups, respectively; in the CCB subgroup, the mean morning home SBP/DBP was 138.5/86.2 and 137.8/85.4 mmHg in each treatment group, respectively. Most baseline characteristics were similar between the esaxerenone and trichlormethiazide groups in both the ARB and CCB subgroups. Although no statistical significance test was conducted, the CCB subgroup had numerically lower baseline values for each BP measurement, UACR, and serum potassium. Baseline characteristics of the PPS are shown in Supplementary Table 1.

**Table 1:** Baseline patient characteristics (full analysis set)

Characteristics	ARB		CCB	
	Esaxerenone *n* = 119	Trichlormethiazide *n* = 116	Esaxerenone *n* = 176	Trichlormethiazide *n* = 174
Sex, male	64 (53.8)	66 (56.9)	85 (48.3)	94 (54.0)
Age, years	66.4 ± 11.0	63.7 ± 11.7	64.5 ± 11.8	65.4 ± 12.4
Weight, kg	65.7 ± 15.0	68.7 ± 14.7	65.6 ± 14.3	65.3 ± 13.2
Body mass index, kg/m^2^	25.2 ± 4.4	26.0 ± 4.7	25.5 ± 4.3	25.2 ± 3.9
Morning home SBP, mmHg	142.4 ± 15.5	141.9 ± 14.7	138.5 ± 14.5	137.8 ± 11.8
Morning home DBP, mmHg	87.8 ± 9.8	88.3 ± 10.5	86.2 ± 9.5	85.4 ± 8.4
Bedtime home SBP, mmHg	135.5 ± 16.9	136.8 ± 16.4	134.1 ± 15.1	132.8 ± 11.9
Bedtime home DBP, mmHg	81.5 ± 10.5	82.1 ± 11.9	81.5 ± 10.4	80.7 ± 10.0
Office SBP, mmHg	145.6 ± 18.3	146.8 ± 16.7	142.5 ± 15.0	140.0 ± 13.4
Office DBP, mmHg	83.1 ± 12.0	86.4 ± 12.8	83.7 ± 11.4	81.4 ± 11.3
NT-proBNP, pg/mL	161.5 ± 467.6	104.3 ± 207.4	74.1 ± 95.4	70.5 ± 86.8
<55	53 (44.5)	55 (47.4)	72 (40.9)	85 (48.9)
55 to <125	17 (14.3)	22 (19.0)	46 (26.1)	50 (28.7)
≥125	26 (21.8)	23 (19.8)	23 (13.1)	18 (10.3)
UACR, mg/gCr	159.7 ± 700.8	143.9 ± 641.4	86.9 ± 239.9	73.0 ± 159.5
<30	80 (67.2)	79 (68.1)	113 (64.2)	108 (62.1)
30 to <300	33 (27.7)	29 (25.0)	50 (28.4)	54 (31.0)
≥300	6 (5.0)	8 (6.9)	13 (7.4)	12 (6.9)
Serum potassium, mEq/L	4.27 ± 0.36	4.28 ± 0.33	4.17 ± 0.34	4.17 ± 0.30
Uric acid, mg/dL	5.48 ± 1.19	5.56 ± 1.21	5.32 ± 1.31	5.29 ± 1.20
eGFR_creat_, mL/min/1.73 m^2^	70.7 ± 16.2	68.5 ± 16.7	72.1 ± 15.4	74.6 ± 17.0
Duration of hypertension, years	5.7 ± 5.0	6.0 ± 5.2	5.1 ± 5.1	5.1 ± 4.9
Complication				
T2DM	53 (44.5)	54 (46.6)	66 (37.5)	61 (35.1)
Dyslipidemia	73 (61.3)	80 (69.0)	115 (65.3)	91 (52.3)
Hyperuricemia	18 (15.1)	25 (21.6)	27 (15.3)	19 (10.9)
Heart failure	15 (12.6)	7 (6.0)	7 (4.0)	9 (5.2)
Dose of esaxerenone at baseline (initial dose), mg				
1.25	52 (43.7)	–	66 (37.5)	–
2.5	67 (56.3)	–	110 (62.5)	–
Dose of esaxerenone at EOT (last dose), mg				
1.25	29 (24.4)	–	36 (20.5)	–
2.5	71 (59.7)	–	103 (58.5)	–
5	19 (16.0)	–	37 (21.0)	–
Dose of trichlormethiazide at baseline (initial dose), mg				
0.25	–	1 (0.9)	–	3 (1.7)
0.5	–	7 (6.0)	–	10 (5.7)
1	–	105 (90.5)	–	157 (90.2)
2	–	3 (2.6)	–	4 (2.3)
Dose of trichlormethiazide at EOT (last dose), mg				
0.25	–	0 (0.0)	–	2 (1.1)
0.5	–	10 (8.6)	–	8 (4.6)
1	–	96 (82.8)	–	148 (85.1)
>1 to ≤2	–	9 (7.8)	–	15 (8.6)
≥3	–	1 (0.9)	–	1 (0.6)

Data are n (%) or mean ± standard deviation

*ARB* angiotensin receptor blocker, *CCB* calcium channel blocker, *DBP* diastolic blood pressure, *eGFR*_*creat*_ creatinine-based estimated glomerular filtration rate, *EOT* end of treatment, *NT-proBNP* N-terminal pro-brain natriuretic peptide, *SBP* systolic blood pressure, *T2DM* type 2 diabetes mellitus, *UACR* urinary albumin-to-creatinine ratio

### BP-lowering effects

**Fig. 1:**
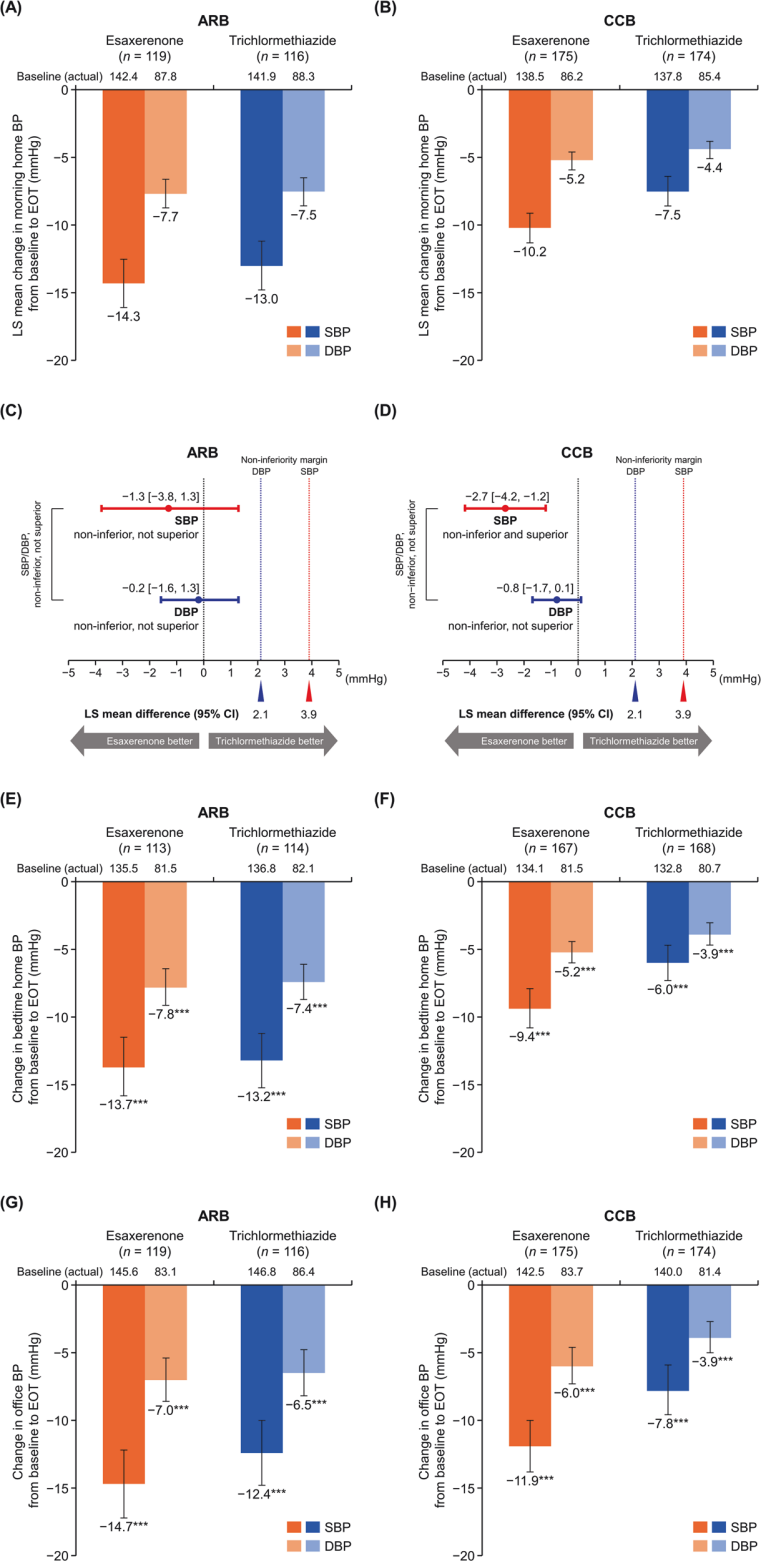
Changes from baseline to EOT in (**A**–**D**) morning home, (**E**, **F**) bedtime home, and (**G**, **H**) office BP (full analysis set). (**A**, **C**, **E**, **G**) ARB subgroup; (**B**, **D**, **F**, **H**) CCB subgroup. For (**C** and **D**), the red dotted line (3.9 mmHg) and blue dotted line (2.1 mmHg) indicate the non-inferiority criteria. Data are LS mean (95% CI) for panels **A**–**D**. Data are arithmetic mean (95% CI) for (**E**–**H**). ***p < 0.001 versus baseline, paired *t*-test. *ARB* angiotensin receptor blocker, *BP* blood pressure, *CCB* calcium channel blocker, *CI* confidence interval, *DBP* diastolic blood pressure, *EOT* end of treatment, *LS* least squares, *SBP* systolic blood pressure

Morning home SBP/DBP significantly decreased from baseline to EOT in all subgroups (Supplementary Table 2). The least squares (LS) mean change in morning home SBP/DBP was −14.3 (95% CI, −16.1, −12.5)/−7.7 (−8.7, −6.6) and −13.0 (−14.8, −11.2)/−7.5 (−8.6, −6.5) mmHg in the esaxerenone and trichlormethiazide groups, respectively, within the ARB subgroup (Fig. 1A) and −10.2 (−11.3, −9.1)/−5.2 (−5.9, −4.6) and −7.5 (−8.6, −6.4)/−4.4 (−5.1, −3.8) mmHg in each treatment group, respectively, within the CCB subgroup (Fig. 1B). The between-group difference in LS mean change was −1.3 (95% CI, −3.8, 1.3)/−0.2 (−1.6, 1.3) mmHg in the ARB subgroup (Fig. 1C) and −2.7 (−4.2, −1.2)/−0.8 (−1.7, 0.1) mmHg in the CCB subgroup (Fig. 1D). The upper limits of the two-sided 95% CIs for SBP and DBP were within the predefined non-inferiority margins in both the ARB and CCB subgroups (non-inferiority margin for SBB/DBP: 3.9/2.1 mmHg). Esaxerenone showed non-inferiority to trichlormethiazide in its antihypertensive effects based on morning home BP regardless of the basal antihypertensive agent used (ARB or CCB). The SBP-lowering effect of esaxerenone was superior to that of trichlormethiazide within the CCB subgroup. Similar results for morning home BP measurement were shown in the PPS (Supplementary Table 3).

Significant reductions from baseline were also shown in bedtime home and office SBP/DBP in both treatment groups in the ARB and CCB subgroups (all *p* < 0.001) (Fig. 1E–H and Supplementary Table 2). Similar results for each BP measurement were shown in the PPS (Supplementary Table 3).

### UACR and NT-proBNP

**Fig. 2:**
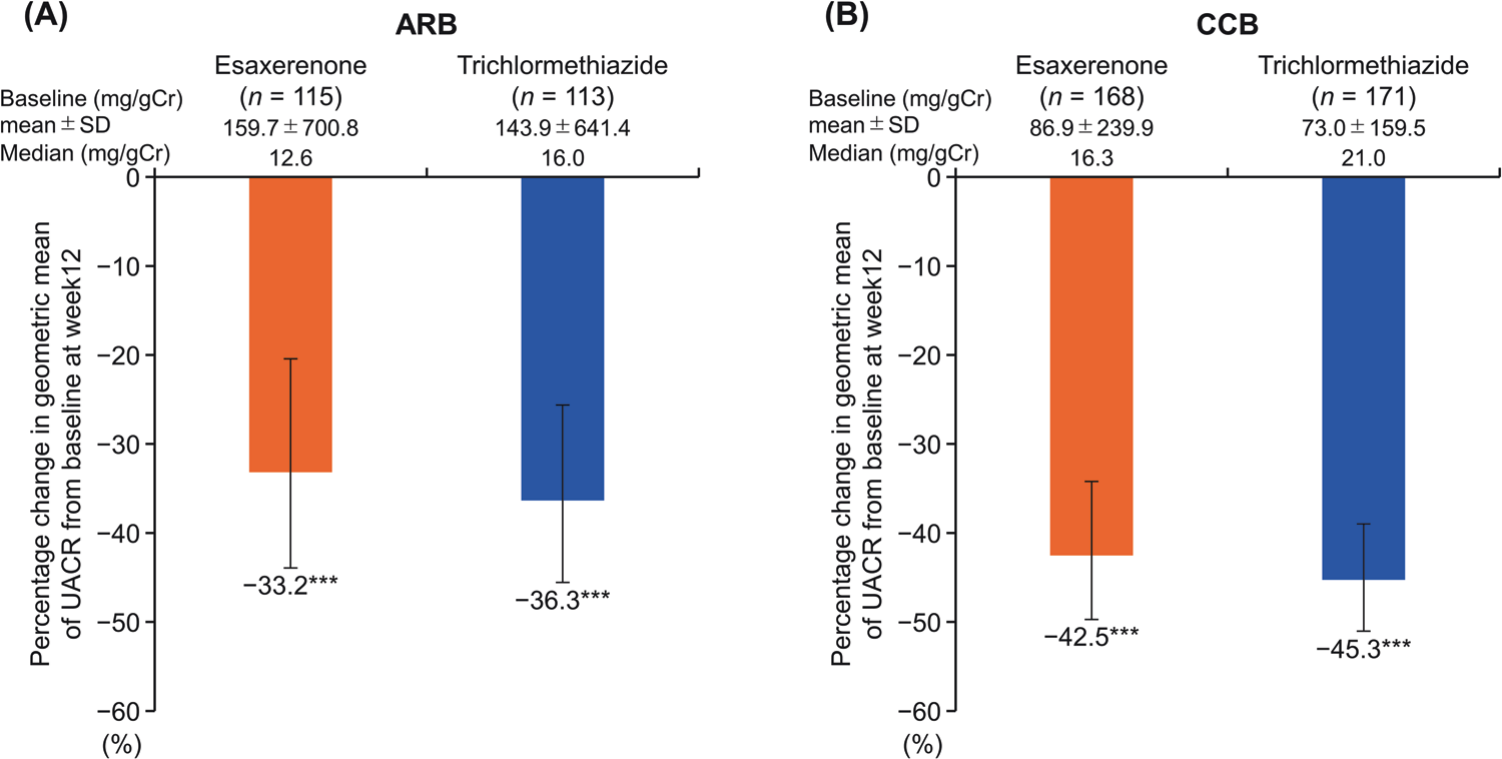
Percentage change in geometric mean of UACR during the study period (full analysis set). (**A**) ARB subgroup; (**B**) CCB subgroup. Data are mean (95% CI). ***p < 0.001 versus baseline, paired *t*-test. *ARB* angiotensin receptor blocker, *CI* confidence interval, *CCB* calcium channel blocker, *SD* standard deviation, *UACR* urinary albumin-to-creatinine ratio

The percentage change in the geometric mean of the UACR significantly decreased from baseline to Week 12 in all subgroups (ARB subgroup: −33.2% and −36.3% in the esaxerenone and trichlormethiazide groups, respectively; CCB subgroup: −42.5% and −45.3% in each treatment group, respectively; all *p* < 0.0001 versus baseline) (Fig. 2, and Supplementary Table 4). Changes in NT-proBNP levels from baseline to Week 12 were −65.86 ± 376.78 pg/mL and −9.95 ± 129.89 pg/mL in the esaxerenone and trichlormethiazide groups within the ARB subgroup, and −11.66 ± 70.29 and −9.25 ± 54.33 in each treatment group, respectively, within the CCB subgroup (Supplementary Table 4). Similar results were also observed in the PPS (Supplementary Table 5).

### eGFR_creat_, serum potassium, and UA

As reported previously [21], overall, treatment-emergent adverse events (TEAEs) occurred in 35.1% and 37.6% of patients in the esaxerenone and trichlormethiazide groups, respectively. Among the TEAEs, blood sodium decreased occurred in 0 (0.0%) patients in the esaxerenone group and three (1.0%) patients in the trichlormethiazide group. Drug-related TEAEs occurred in 6.0% and 9.4% of patients in each treatment group, respectively. Most TEAEs were mild or moderate in both the esaxerenone and trichlormethiazide groups. Serious TEAEs were reported in three (1.0%) patients in each treatment group, and none were related to the study drugs. This subgroup analysis focused on eGFR_creat_, serum potassium, and UA; TEAEs were not analyzed in the CCB and ARB subgroups according to the prespecified analysis outlined in the clinical trial protocol.

**Fig. 3:**
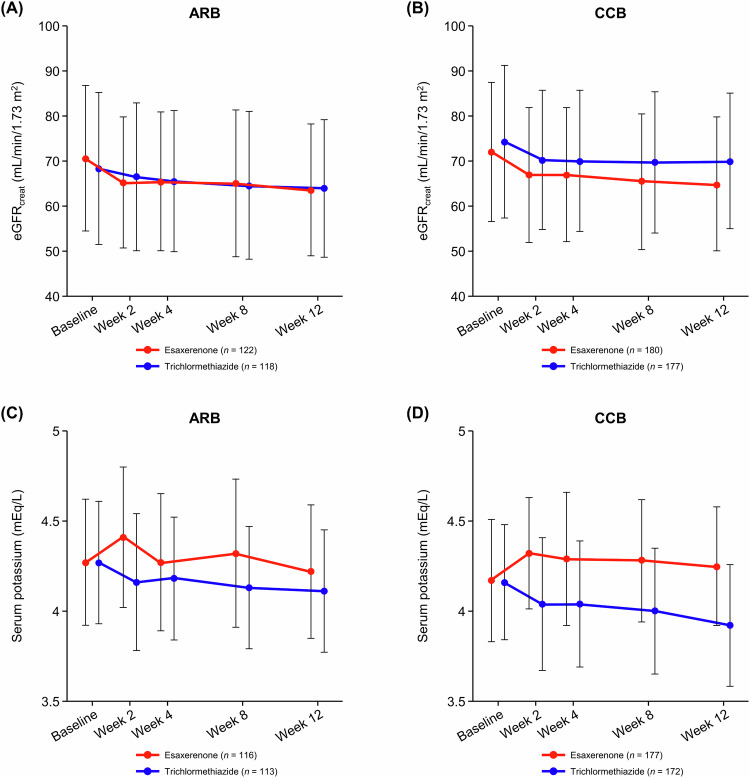
Time course changes in eGFR_creat_ (**A**, **B**) and serum potassium levels (**C**, **D**) during the study period (safety analysis set). (**A**, **C**) ARB subgroup; (**B**, **D**) CCB subgroup. Data are mean ± standard deviation. *ARB* angiotensin receptor blocker, *CCB* calcium channel blocker, *eGFR*_*creat*_ creatinine-based estimated glomerular filtration rate

Time course changes in eGFR_creat_ and serum potassium are shown in Fig. 3. eGFR_creat_ decreased over the first 2 weeks of treatment and remained stable until Week 12 (Fig. 3A, B, and Supplementary Table 6). The change in eGFR_creat_ from baseline to Week 12 was −7.06 ± 8.31 and −4.74 ± 9.02 mL/min/1.73 m^2^ in the esaxerenone and trichlormethiazide groups, respectively, within the ARB subgroup, and −7.10 ± 8.84 and −4.63 ± 10.37 mL/min/1.73 m^2^ in each treatment group, respectively, within the CCB subgroup.

Serum potassium levels increased over the first 2 weeks after starting esaxerenone treatment in both the ARB and CCB subgroups, then decreased up to Week 4, and remained stable until Week 12 in the ARB subgroup (Fig. 3C); whereas after the first 2 weeks, serum potassium levels gradually decreased up to Week 12 in the CCB subgroup (Fig. 3D and Supplementary Table 6). Serum potassium levels gradually decreased in the trichlormethiazide group irrespective of the basal antihypertensive agent used (ARB or CCB).

The respective percentages of patients with serum potassium levels <3.5 mEq/L and ≥5.5 mEq/L in the ARB subgroup were 3.4% and 4.2% for esaxerenone and 7.9% and 0% for trichlormethiazide; the respective percentages were 2.8% and 0.6% for esaxerenone and 13.9% and 1.2% for trichlormethiazide in the CCB subgroup (Supplementary Table 7). Serum potassium levels ≥6.0 mEq/L were not observed in any of the subgroups.

The percentages of patients with UA levels ≥7.0 mg/dL were numerically higher in the trichlormethiazide group than the esaxerenone group in both the ARB and CCB subgroups (ARB subgroup: esaxerenone, 27.9% and trichlormethiazide, 39.5%; CCB subgroup: esaxerenone, 27.2%, trichlormethiazide, 31.3%; Supplementary Table 8).

## Discussion

### Summary of results

Our subgroup analysis, prespecified in the statistical analysis plan of the EXCITE-HT study, aimed to investigate the comparative efficacy of esaxerenone and trichlormethiazide as a second-line antihypertensive agent, based on the basal antihypertensive agent used (ARB or CCB). CCBs were used more frequently than ARBs as a basal antihypertensive agent among patients enrolled in this study (ARBs in approximately 40%; CCBs in approximately 60%). The proportion of patients with type 2 diabetes mellitus (T2DM) was approximately 45% among those in the ARB subgroup and approximately 36% among those in the CCB subgroup. We found that esaxerenone was not inferior to trichlormethiazide in lowering morning home BP, irrespective of the basal antihypertensive agent used. In the ARB subgroup, esaxerenone was non-inferior to trichlormethiazide in lowering both SBP and DBP. In the CCB subgroup, esaxerenone was superior to trichlormethiazide in lowering SBP and non-inferior in lowering DBP. The same trends were observed not only for morning home BP but also for bedtime home and office BP. In the esaxerenone group, a higher percentage of patients in the ARB subgroup (4.2%) than in the CCB subgroup (0.6%) had serum potassium levels ≥5.5 mEq/L. However, no patient in either subgroup exhibited serum potassium levels ≥6.0 mEq/L. In the trichlormethiazide group, the percentage of patients with serum potassium levels <3.5 mEq/L was higher in the CCB subgroup than the ARB subgroup (13.9% and 7.9%, respectively).

### Antihypertensive effects in combination with a basal antihypertensive agent

The antihypertensive effect of esaxerenone was superior to that of trichlormethiazide for SBP and non-inferior to that of trichlormethiazide for DBP in the CCB subgroup. The change in BP from baseline to EOT was smaller in the CCB subgroup than the ARB subgroup in both the esaxerenone and trichlormethiazide groups. However, baseline SBP/DBP was lower by approximately 4/2 mmHg in the CCB subgroup versus the ARB subgroup, and SBP/DBP at EOT was comparable between the ARB and CCB subgroups in the esaxerenone group. Therefore, esaxerenone is considered to have a significant antihypertensive effect regardless of the basal antihypertensive agent used (ARB or CCB).

In contrast, the antihypertensive effect of trichlormethiazide was less potent in the CCB subgroup than in the ARB subgroup, even when differences in baseline values were taken into account. The combination of an ARB and trichlormethiazide is thought to exert a synergistic antihypertensive effect. The antihypertensive effect of ARBs is attenuated by excessive salt intake and may be inadequate [25], while diuretics inhibit Na^+^/Cl^−^ co-transporters in the distal tubule, reducing Na^+^ reabsorption and improving salt sensitivity. One potential explanation for this differential effect may be the phenomenon of aldosterone breakthrough, which was observed in 30% to 50% of patients with hypertension undergoing long-term treatment with RAS inhibitors, in whom the production of aldosterone increased as a compensatory mechanism, leading to higher plasma aldosterone levels [26, 27]. Aldosterone breakthrough may have been more prevalent in patients taking RAS inhibitors than in those taking CCBs. There are now single-pill combinations of diuretics and ARBs on the market, and the combination of these two agents is recommended in the JSH 2019 guideline [8].

Interestingly, our study showed that the antihypertensive effect of esaxerenone was non-inferior to that of trichlormethiazide when added to an ARB, and that of esaxerenone was consistent with the findings of the ESAX-HTN and ENaK studies [19, 28]. In addition, a pooled analysis of five clinical studies of esaxerenone [29], reported that the combination with a RAS inhibitor contributed to a stronger antihypertensive effect of esaxerenone compared with the combination with a CCB, aligning with our study results.

The mechanism responsible for the stronger antihypertensive effect of esaxerenone when co-administered with an ARB than CCB is unclear. Conditions such as obesity, T2DM, chronic kidney disease (including diabetic kidney disease), excessive salt intake, and aldosterone breakthrough [30, 31], which have been suggested as factors affecting the antihypertensive effect of esaxerenone [28, 32], could possibly explain this observation. In the present study, the higher percentage of patients with T2DM as a complication (44.5% for ARBs and 37.5% for CCBs), the higher mean UACR (159.7 mg/g Cr for ARB and 86.9 mg/gCr for CCB), and the higher mean NT-proBNP (161.5 pg/mL for ARB and 74.1 pg/mL for CCB) in the ARB subgroup than the CCB subgroup among patients treated with esaxerenone suggests that the ARB subgroup may have included more patients with diabetic kidney disease. This may have influenced the difference in antihypertensive effect between the ARB and CCB subgroups among patients treated with esaxerenone.

Patients with inadequate antihypertensive response to ARBs in clinical practice were included in this study, and the duration of hypertension was longer in the ARB subgroup (esaxerenone, 5.7 ± 5.0; trichlormethiazide, 6.0 ± 5.2 years) than in the CCB subgroup (esaxerenone, 5.1 ± 5.1; trichlormethiazide, 5.1 ± 4.9 years). One possible explanation is that a certain number of patients with aldosterone breakthrough may have been included, which could have also influenced the stronger antihypertensive effect of esaxerenone in patients taking ARBs than in those taking CCBs. However, it should be noted that, in this study, plasma aldosterone concentration and plasma renin activity were not measured, and data on the duration of ARB and CCB use were not collected. Another possible explanation may be that the antihypertensive effect of esaxerenone was weaker with a CCB than with an ARB because of the diuretic effect of CCBs [33], despite a larger proportion of patients receiving esaxerenone at the maximum dose (5 mg) when combined with a CCB than with an ARB. It remains to be clarified whether the antihypertensive effect of esaxerenone is stronger when combined with an ARB than with a CCB. Nevertheless, the fact that esaxerenone can be expected to have stronger BP-lowering effects than diuretics in patients with inadequately controlled hypertension despite long-term treatment with ARBs is an important finding regarding the selection of second-line agents for hypertensive patients.

### UACR

The improvement in UACR was similar between the esaxerenone and trichlormethiazide groups, with no significant difference based on basal antihypertensive agent at Week 12. However, it should be noted that the results are simply observations of the change in UACR over 12 weeks when esaxerenone or trichlormethiazide were added to the basal antihypertensive agent. Although the baseline UACR was higher in the ARB subgroup than the CCB subgroup, it is assumed that the UACR-lowering effect of these drugs had reached a steady state in this study because these basal antihypertensive agents were pre-administered for ≥4 weeks prior to registration. Therefore, the UACR-lowering effect of esaxerenone and trichlormethiazide when co-administered as add-on to an ARB or CCB may have been underestimated. To clarify the difference in UACR improvement between esaxerenone and trichlormethiazide and between ARB and CCB, an analysis by baseline value, simultaneous administration of basal and second-line antihypertensive agent from the first dosing time, a larger sample size, and long-term observation will be necessary.

### Serum potassium

Overall, no incidence of serum potassium level ≥6.0 mEq/L was observed, although the risk of hyperkalemia with esaxerenone and risk of hypokalemia with trichlormethiazide are known class effects [22, 34]. The percentage of patients with serum potassium level ≥5.5 mEq/L was higher in the esaxerenone group than the trichlormethiazide group, but the incidence was lower than in other studies of esaxerenone [22, 28, 32, 35–37]. The incidence of serum potassium level ≥5.5 mEq/L was higher in the ARB subgroup than the CCB subgroup (esaxerenone: ARB, 4.2% and CCB, 0.6%), but the ARB subgroup had lower eGFR and higher UACR than the CCB subgroup, suggesting that more patients with impaired renal function were in the ARB subgroup. Since renal dysfunction, as well as ARB use, are known risk factors for hyperkalemia [22], our results do not necessarily suggest that esaxerenone is less compatible with ARBs than CCBs. In fact, there was no notable difference in serum potassium levels at 12 weeks between the ARB and CCB subgroups (esaxerenone: 4.22 ± 0.37 and 4.25 ± 0.33 mEq/L, respectively). Thus, even with the addition of esaxerenone to an ARB, serum potassium elevation can be managed in the same way when added to a CCB by dose reduction and titration of esaxerenone along with regular serum potassium monitoring according to the package insert. The serum potassium trend peaked at Week 2 and decreased thereafter, suggesting that monitoring in the early stages of administration may be more important.

The trichlormethiazide group had a higher frequency of low serum potassium level (<3.5 mEq/L) than the esaxerenone group, and its frequency was higher in the CCB subgroup than the ARB subgroup (13.9% versus 7.9%, respectively). ARBs increase serum potassium, whereas CCBs are thought to have no effect on serum potassium. The effects on serum potassium may have been offset by the combination of ARBs and trichlormethiazide.

In the EXCITE-HT study, the incidence of blood sodium decreased was 0% in the esaxerenone group and 1% in the trichlormethiazide group [21]. Hyponatremia is noted as an adverse effect of unknown frequency in the esaxerenone package insert [23] and as a serious adverse effect in the trichlormethiazide package insert [24].

### Uric acid

Within the esaxerenone group, no significant difference was observed in UA elevations between the ARB and CCB subgroups. These elevations were less frequent compared with trichlormethiazide. Notably, within the trichlormethiazide group, the percentage of patients with UA ≥ 7.0 mg/dL was numerically higher when used in combination with an ARB than a CCB (ARB 39.5%, CCB 31.3%). Elevated serum UA levels are associated with increased cardiovascular mortality, thus maintaining optimal serum UA levels is crucial in preventing cardiovascular events [38]. Losartan [39–41] and cilnidipine (a CCB) [42] are known to have UA-lowering effects, but this study did not analyze by type of basal antihypertensive agent, so this warrants further investigation.

### Clinical implications

The EXCITE-HT study demonstrated the efficacy and safety of esaxerenone as a second-line antihypertensive agent. To our knowledge, no prior studies have reported the differences in efficacy and safety of different basal antihypertensive agents when MRBs are co-administered, and this is the first report of its kind. The present study was conducted in a clinical setting, and the ARB:CCB ratio was 2:3 in patients with inadequate antihypertensive control treatment with a single agent. This suggests that the number of patients with inadequate antihypertensive control with single agents in Japanese clinical practice may be slightly higher with CCBs than ARBs. Furthermore, adding esaxerenone as a second-line agent in patients with uncontrolled hypertension receiving CCB monotherapy may be expected to have a stronger antihypertensive effect compared with diuretics, with fewer safety concerns such as UA elevation and hypokalemia risk. For patients with an inadequate antihypertensive effect with an ARB or CCB, the addition of esaxerenone or trichlormethiazide may be a better option in terms of BP control than the continuation of ARB or CCB monotherapy at higher doses.

Introducing esaxerenone in patients on ARB monotherapy can be expected to provide a more potent antihypertensive effect than when added to a CCB. When esaxerenone is combined with an ARB, more attention should be paid to serum potassium elevation compared with when it is added to a CCB. Adherence to package inserts—starting at small doses and titrating with careful monitoring—is important for safe use.

To date, few studies have focused on combination therapy with different drug classes, and this study provides valuable evidence for such combinations. We anticipate further research into the positioning of esaxerenone as a concomitant agent, which could potentially decrease the number of patients with inadequate hypertension control.

### Limitations

The study limitations are similar to those of the main study [20, 21]. ARBs and CCBs were not randomly assigned to patients; these were prescribed based on the physicians’ judgment under real clinical setting to suit their patients, which may have led to bias. There were differences between the ARB and CCB subgroups in some patient characteristics and baseline BP measurements, and these differences were not adjusted for in this study. The analysis of ARBs and CCBs by individual drug, rather than by drug class, was not performed, and the unique characteristics reported for some ARBs and CCBs could not be taken into account. The combination of ARBs and CCBs represents the most common two-drug combination in real-world clinical practice. From the present study, the usefulness (efficacy and safety) of the combination of esaxerenone with an ARB or CCB versus the combination of ARBs and CCBs has not been clarified, and this remains an important clinical question. Of note, from the pathophysiological point of view (e.g., the aldosterone breakthrough phenomenon), combination treatment with esaxerenone plus an ARB might provide a greater benefit compared with esaxerenone plus a CCB. However, in the present study, no statistical tests were performed to determine whether there are differences in the antihypertensive effect of esaxerenone plus an ARB vs esaxerenone plus a CCB, and the study duration was short (12 weeks). A longer-term study is necessary to clarify this in the future. Finally, to consider esaxerenone as a first-line treatment option for hypertension, it is essential to demonstrate a reduction in cardiovascular events through the BP-lowering effect of esaxerenone. However, the study duration was short, and the sample size may have been insufficient to assess the prognosis for cardiovascular events and mortality. The results of this study and the main analysis paper support the use of esaxerenone as a second-line treatment and suggest its potential to become a second-line agent.

## Conclusion

The non-inferiority of esaxerenone to trichlormethiazide in lowering morning home BP was shown regardless of whether the basal antihypertensive agent was an ARB or a CCB. Adding esaxerenone to a CCB showed superiority to trichlormethiazide in lowering SBP and non-inferiority to trichlormethiazide in lowering DBP. Serum potassium was more likely to be elevated when esaxerenone was added to an ARB than to a CCB. However, such adverse effects of esaxerenone can be mitigated if administered according to the package insert.

## Supplementary information

Supplementary materials

## Data Availability

The anonymized data underlying the results presented in this manuscript may be made available to researchers upon submission of a reasonable request to the corresponding author. The decision to disclose the data will be made by the corresponding author and the funder, Daiichi Sankyo Co., Ltd. Data disclosure can be requested for 36 months from article publication.
