# Supplementary material for: Home blood pressure-lowering effect of esaxerenone versus trichlormethiazide for uncontrolled hypertension: a predefined subanalysis of the EXCITE-HT randomized controlled trial by basal calcium channel blocker versus angiotensin receptor blocker
Source: Hypertens Res. 2024 Oct 12;48(2):506–18. doi: 10.1038/s41440-024-01887-1 (PMC11794140; doi:10.1038/s41440-024-01887-1)
Supplement: Supplementary file 1 — Supplementary materials [file 41440_2024_1887_MOESM1_ESM.docx]

**Home blood pressure-lowering effect of esaxerenone versus trichlormethiazide for uncontrolled hypertension: a predefined subanalysis of the EXCITE-HT randomized controlled trial by basal calcium channel blocker versus angiotensin receptor blocker**

Kazuomi Kario, Hiroyuki Ohbayashi, Masami Hashimoto, Naoki Itabashi, Mitsutoshi Kato, Kazuaki Uchiyama, Kunio Hirano, Noriko Nakamura, Takahide Miyamoto, Hirotaka Nagashima, Hidenori Ishida, Yusuke Ebe, Tsuguru Hatta, Toshiki Fukui, Tatsuo Shimosawa, Tomohiro Katsuya, Takashi Taguchi, Ayumi Tanabe, and Mitsuru Ohishi, on behalf of the EXCITE-HT investigators

**Supplementary Table 1**. Baseline patient characteristics (per protocol set)

|  | **ARB** | | **CCB** | |
| --- | --- | --- | --- | --- |
|  | **Esaxerenone**  ***n* = 108** | **Trichlormethiazide**  ***n* = 116** | **Esaxerenone**  ***n* = 167** | **Trichlormethiazide**  ***n* = 174** |
| Sex, male | 61 (56.5) | 66 (56.9) | 82 (49.1) | 94 (54.0) |
| Age, years | 66.8 ± 11.1 | 63.7 ± 11.7 | 63.9 ± 11.8 | 65.4 ± 12.4 |
| Weight, kg | 65.2 ± 14.9 | 68.7 ± 14.7 | 65.7 ± 14.4 | 65.3 ± 13.2 |
| Body mass index, kg/m^2^ | 25.0 ± 4.0 | 26.0 ± 4.7 | 25.5 ± 4.3 | 25.2 ± 3.9 |
| Morning home SBP, mmHg | 143.3 ± 15.9 | 141.9 ± 14.7 | 139.0 ± 14.5 | 137.8 ± 11.8 |
| Morning home DBP, mmHg | 87.9 ± 10.0 | 88.3 ± 10.5 | 86.5 ± 9.6 | 85.4 ± 8.4 |
| Bedtime home SBP, mmHg | 136.3 ± 17.3 | 136.8 ± 16.4 | 134.7 ± 15.2 | 132.8 ± 11.9 |
| Bedtime home DBP, mmHg | 81.6 ± 10.8 | 82.1 ± 11.9 | 81.9 ± 10.5 | 80.7 ± 10.0 |
| Office SBP, mmHg | 145.8 ± 18.6 | 146.8 ± 16.7 | 142.1 ± 15.0 | 140.0 ± 13.4 |
| Office DBP, mmHg | 82.9 ± 12.0 | 86.4 ± 12.8 | 83.8 ± 11.5 | 81.4 ± 11.3 |
| NT-proBNP, pg/mL | 168.5 ± 484.9 | 104.3 ± 207.4 | 72.5 ± 95.9 | 70.5 ± 86.8 |
| <55 | 51 (47.2) | 55 (47.4) | 69 (41.3) | 85 (48.9) |
| 55 to <125 | 13 (12.0) | 22 (19.0) | 45 (26.9) | 50 (28.7) |
| ≥125 | 25 (23.1) | 23 (19.8) | 20 (12.0) | 18 (10.3) |
| UACR, mg/gCr | 158.8 ± 725.4 | 143.9 ± 641.4 | 90.6 ± 245.8 | 73.0 ± 159.5 |
| <30 | 73 (67.6) | 79 (68.1) | 105 (62.9) | 108 (62.1) |
| 30 to <300 | 30 (27.8) | 29 (25.0) | 49 (29.3) | 54 (31.0) |
| ≥300 | 5 (4.6) | 8 (6.9) | 13 (7.8) | 12 (6.9) |
| Serum potassium, mEq/L | 4.27 ± 0.36 | 4.28 ± 0.33 | 4.16 ± 0.34 | 4.17 ± 0.30 |
| Uric acid, mg/dL | 5.54 ± 1.19 | 5.56 ± 1.21 | 5.32 ± 1.32 | 5.29 ± 1.20 |
| eGFR_creat_, mL/min/1.73 m^2^ | 70.81 ± 16.58 | 68.49 ± 16.67 | 72.39 ± 15.46 | 74.57 ± 16.99 |
| Duration of hypertension, years | 5.87 ± 5.06 | 6.04 ± 5.18 | 4.91 ± 5.05 | 5.09 ± 4.92 |
| Complication | 104 (96.3) | 113 (97.4) | 162 (97.0) | 163 (93.7) |
| T2DM | 47 (43.5) | 54 (46.6) | 59 (35.3) | 61 (35.1) |
| Dyslipidemia | 68 (63.0) | 80 (69.0) | 108 (64.7) | 91 (52.3) |
| Hyperuricemia | 16 (14.8) | 25 (21.6) | 27 (16.2) | 19 (10.9) |
| Heart failure | 13 (12.0) | 7 (6.0) | 6 (3.6) | 9 (5.2) |
| Dose of esaxerenone at baseline (initial dose), mg |  |  |  |  |
| 1.25 | 44 (40.7) | - | 60 (35.9) | - |
| 2.5 | 64 (59.3) | - | 107 (64.1) | - |
| Dose of esaxerenone at EOT (last dose), mg |  |  |  |  |
| 1.25 | 24 (22.2) | - | 31 (18.6) | - |
| 2.5 | 65 (60.2) | - | 100 (59.9) | - |
| 5 | 19 (17.6) | - | 36 (21.6) | - |
| Dose of trichlormethiazide at baseline (initial dose), mg |  |  |  |  |
| 0.25 | - | 1 (0.9) | - | 3 (1.7) |
| 0.5 | - | 7 (6.0) | - | 10 (5.7) |
| 1 | - | 105 (90.5) | - | 157 (90.2) |
| 2 | - | 3 (2.6) | - | 4 (2.3) |
| Dose of trichlormethiazide at EOT (last dose), mg |  |  |  |  |
| 0.25 | - | 0 (0.0) | - | 2 (1.1) |
| 0.5 | - | 10 (8.6) | - | 8 (4.6) |
| 1 | - | 96 (82.8) | - | 148 (85.1) |
| >1 to ≤2 | - | 9 (7.8) | - | 15 (8.6) |
| ≥3 | - | 1 (0.9) | - | 1 (0.6) |

Data are n (%) or mean ± standard deviation.

*ARB* angiotensin receptor blocker, *CCB* calcium channel blocker, *DBP* diastolic blood pressure, *eGFR_creat_* creatinine-based estimated glomerular filtration rate, *EOT* end of treatment, *NT-proBNP* N-terminal pro-brain natriuretic peptide, *SBP* systolic blood pressure, *T2DM* type 2 diabetes mellitus, *UACR* urinary albumin-to-creatinine ratio.

**Supplementary Table 2.** Change from baseline in BP (full analysis set)

|  | **ARB** | | | | | | **CCB** | | | | | |
| --- | --- | --- | --- | --- | --- | --- | --- | --- | --- | --- | --- | --- |
|  | **Esaxerenone** | | | **Trichlormethiazide** | | | **Esaxerenone** | | | **Trichlormethiazide** | | |
|  | ***n*** | **SBP, mmHg** | **DBP, mmHg** | ***n*** | **SBP, mmHg** | **DBP, mmHg** | ***n*** | **SBP, mmHg** | **DBP, mmHg** | ***n*** | **SBP, mmHg** | **DBP, mmHg** |
| Morning home BP, mmHg |  |  |  |  |  |  |  |  |  |  |  |  |
| Baseline | 119 | 142.4 ± 15.5 | 87.8 ± 9.8 | 116 | 141.9 ± 14.7 | 88.3 ± 10.5 | 176 | 138.5 ± 14.5 | 86.2 ± 9.5 | 174 | 137.8 ± 11.8 | 85.4 ± 8.4 |
| Week 2 | 119 | 134.4 ± 16.0 | 84.0 ± 9.6 | 116 | 133.1 ± 14.7 | 83.7 ± 10.4 | 176 | 132.8 ± 13.8 | 83.6 ± 9.2 | 174 | 133.5 ± 11.4 | 83.4 ± 8.7 |
| Change from baseline | 119 | −8.0 ± 8.6*** | −3.8 ± 5.0*** | 116 | −8.8 ± 9.7*** | −4.5 ± 5.1*** | 176 | −5.6 ± 6.7*** | −2.6 ± 3.8*** | 174 | −4.3 ± 7.1*** | −2.0 ± 4.1*** |
| Week 4 | 119 | 131.4 ± 16.1 | 82.1 ± 9.4 | 116 | 131.8 ± 14.7 | 82.9 ± 10.0 | 176 | 131.5 ± 13.9 | 82.6 ± 9.4 | 174 | 132.9 ± 12.1 | 82.9 ± 8.8 |
| Change from baseline | 119 | −11.0 ± 9.9*** | −5.7 ± 5.4*** | 116 | −10.1 ± 9.5*** | −5.4 ± 5.0*** | 176 | −7.0 ± 7.3*** | −3.6 ± 4.2*** | 174 | −4.9 ± 7.6*** | −2.5 ± 4.0*** |
| Week 6 | 118 | 129.9 ± 15.6 | 81.1 ± 9.6 | 115 | 130.6 ± 14.3 | 81.7 ± 9.9 | 173 | 130.7 ± 14.4 | 82.0 ± 9.2 | 174 | 132.2 ± 12.1 | 82.3 ± 8.7 |
| Change from baseline | 118 | −12.7 ± 10.1*** | −6.8 ± 5.8*** | 115 | −11.3 ± 9.7*** | −6.5 ± 5.6*** | 173 | −7.7 ± 7.2*** | −4.2 ± 4.1*** | 174 | −5.6 ± 8.3*** | −3.1 ± 4.3*** |
| Week 8 | 117 | 130.1 ± 15.0 | 81.3 ± 9.6 | 115 | 130.1 ± 14.8 | 81.7 ± 10.4 | 172 | 130.4 ± 14.0 | 82.0 ± 9.3 | 173 | 131.2 ± 11.6 | 81.4 ± 8.8 |
| Change from baseline | 117 | −12.6 ± 10.2*** | −6.5 ± 6.3*** | 115 | −11.8 ± 11.0*** | −6.5 ± 5.7*** | 172 | −7.9 ± 7.6*** | −4.1 ± 4.4*** | 173 | −6.7 ± 8.4*** | −4.1 ± 4.5*** |
| Week 10 | 115 | 127.7 ± 14.7 | 80.0 ± 9.4 | 113 | 129.3 ± 14.3 | 81.3 ± 10.6 | 171 | 128.8 ± 13.0 | 80.9 ± 9.4 | 172 | 130.9 ± 11.1 | 81.4 ± 8.9 |
| Change from baseline | 115 | −14.9 ± 11.8*** | −7.9 ± 6.3*** | 113 | −12.3 ± 9.6*** | −6.8 ± 5.0*** | 171 | −9.4 ± 8.7*** | −5.2 ± 5.0*** | 172 | −7.0 ± 8.6*** | −4.0 ± 4.6*** |
| Week 12 | 115 | 128.2 ± 14.2 | 80.0 ± 9.7 | 113 | 129.2 ± 14.0 | 80.9 ± 10.9 | 168 | 127.9 ± 12.2 | 80.7 ± 9.2 | 172 | 130.6 ± 10.7 | 81.1 ± 8.6 |
| Change from baseline | 115 | −14.4 ± 11.9*** | −7.8 ± 6.5*** | 113 | −12.4 ± 10.4*** | −7.2 ± 6.4*** | 168 | −10.3 ± 9.0*** | −5.4 ± 4.8*** | 172 | −7.4 ± 7.5*** | −4.4 ± 4.1*** |
| LS mean change from baseline [95% CI] |  | −14.1  [−15.9, −12.3] | −7.6  [−8.7, −6.5] |  | −12.8  [−14.6, −10.9] | −7.4  [−8.5, −6.3] |  | −10.4  [−11.4, −9.3] | −5.4  [−6.1, −4.8] |  | −7.4  [−8.4, −6.3] | −4.4  [−5.0, −3.8] |
| Difference in LS mean change from baseline [95% CI] |  | −1.3  [−3.9, 1.3] | −0.2  [−1.7, 1.3] |  | - | - |  | −3.0  [−4.5, −1.5] | −1.0  [−1.9, −0.1] |  | - | - |
| EOT | 119 | 127.9 ± 14.2 | 79.9 ± 9.6 | 116 | 129.2 ± 13.9 | 80.9 ± 10.7 | 175 | 128.2 ± 12.5 | 80.9 ± 9.2 | 174 | 130.4 ± 10.9 | 81.0 ± 8.6 |
| Change from baseline | 119 | −14.5 ± 11.9*** | −7.9 ± 6.4*** | 116 | −12.7 ± 10.6*** | −7.3 ± 6.4*** | 175 | −10.2 ± 9.2*** | −5.3 ± 5.0*** | 174 | −7.4 ± 7.5*** | −4.4 ± 4.1*** |
| LS mean change from baseline [95% CI] |  | −14.3  [−16.1, −12.5] | −7.7  [−8.7, −6.6] |  | −13.0  [−14.8, −11.2] | −7.5  [−8.6, −6.5] |  | −10.2  [−11.3, −9.1] | −5.2  [−5.9, −4.6] |  | −7.5  [−8.6, −6.4] | −4.4  [−5.1, −3.8] |
| Difference in LS mean change from baseline [95% CI] |  | −1.3  [−3.8, 1.3] | −0.2  [−1.6, 1.3] |  | - | - |  | −2.7  [−4.2, −1.2] | −0.8  [−1.7, 0.1] |  | - | - |
| Bedtime home BP, mmHg |  |  |  |  |  |  |  |  |  |  |  |  |
| Baseline | 113 | 135.5 ± 16.9 | 81.5 ± 10.5 | 114 | 136.8 ± 16.4 | 82.1 ± 11.9 | 168 | 134.1 ± 15.1 | 81.5 ± 10.4 | 168 | 132.8 ± 11.9 | 80.7 ± 10.0 |
| Week 2 | 118 | 127.9 ± 17.8 | 78.1 ± 11.1 | 116 | 128.1 ± 15.1 | 77.8 ± 11.2 | 171 | 129.0 ± 13.8 | 79.5 ± 9.6 | 172 | 129.6 ± 11.7 | 78.9 ± 9.5 |
| Change from baseline | 113 | −8.2 ± 9.0*** | −3.5 ± 4.9*** | 114 | −8.6 ± 8.4*** | −4.3 ± 4.7*** | 168 | −5.1 ± 7.3*** | −2.0 ± 4.1*** | 168 | −3.5 ± 7.0*** | −1.8 ± 4.7*** |
| Week 4 | 118 | 125.4 ± 17.0 | 76.1 ± 10.2 | 116 | 126.4 ± 14.7 | 76.8 ± 10.6 | 174 | 127.6 ± 13.7 | 78.4 ± 10.0 | 172 | 129.0 ± 11.7 | 78.4 ± 9.3 |
| Change from baseline | 113 | −10.9 ± 9.9*** | −5.7 ± 5.3*** | 114 | −10.2 ± 9.2*** | −5.3 ± 5.6*** | 168 | −6.7 ± 8.0*** | −3.2 ± 4.9*** | 168 | −4.1 ± 7.8*** | −2.3 ± 5.3*** |
| Week 6 | 117 | 125.0 ± 16.7 | 76.0 ± 10.9 | 115 | 125.0 ± 14.9 | 75.9 ± 10.7 | 171 | 126.2 ± 13.3 | 77.5 ± 9.5 | 172 | 127.9 ± 11.0 | 77.7 ± 9.2 |
| Change from baseline | 112 | −11.2 ± 10.7*** | −5.7 ± 6.2*** | 113 | −11.8 ± 9.9*** | −6.2 ± 6.3*** | 165 | −8.1 ± 8.6*** | −4.2 ± 4.9*** | 168 | −5.1 ± 7.6*** | −2.9 ± 5.3*** |
| Week 8 | 116 | 124.0 ± 16.2 | 75.0 ± 10.4 | 115 | 124.5 ± 14.6 | 75.6 ± 10.8 | 170 | 125.7 ± 13.1 | 77.1 ± 9.9 | 171 | 127.5 ± 11.6 | 77.2 ± 9.3 |
| Change from baseline | 111 | −12.2 ± 10.1*** | −6.6 ± 6.4*** | 113 | −12.2 ± 11.0*** | −6.5 ± 6.6*** | 164 | −8.3 ± 8.7*** | −4.4 ± 5.1*** | 167 | −5.6 ± 8.0*** | −3.5 ± 5.0*** |
| Week 10 | 114 | 122.2 ± 15.5 | 74.4 ± 9.8 | 113 | 122.9 ± 15.5 | 74.5 ± 11.0 | 169 | 125.0 ± 11.4 | 76.9 ± 9.4 | 170 | 127.6 ± 11.5 | 76.7 ± 9.7 |
| Change from baseline | 109 | −14.4 ± 11.7*** | −7.5 ± 6.8*** | 111 | −13.4 ± 11.1*** | −7.4 ± 6.5*** | 163 | −9.0 ± 9.6*** | −4.8 ± 5.5*** | 166 | −5.7 ± 8.5*** | −4.0 ± 5.6*** |
| Week 12 | 114 | 122.6 ± 15.1 | 74.0 ± 10.3 | 113 | 123.6 ± 15.8 | 74.6 ± 11.3 | 166 | 124.5 ± 11.9 | 76.4 ± 9.6 | 170 | 127.2 ± 11.5 | 76.8 ± 9.0 |
| Change from baseline | 109 | −13.9 ± 11.5*** | −7.9 ± 7.0*** | 111 | −12.9 ± 10.9*** | −7.3 ± 7.0*** | 160 | −9.5 ± 9.4*** | −5.4 ± 5.2*** | 166 | −6.0 ± 8.5*** | −3.9 ± 5.6*** |
| EOT | 118 | 122.3 ± 15.1 | 73.9 ± 10.3 | 116 | 123.6 ± 15.6 | 74.7 ± 11.2 | 173 | 124.8 ± 12.2 | 76.4 ± 9.4 | 172 | 127.0 ± 11.6 | 76.8 ± 9.0 |
| Change from baseline | 113 | −13.7 ± 11.6*** | −7.8 ± 7.0*** | 114 | −13.2 ± 10.9*** | −7.4 ± 7.0*** | 167 | −9.4 ± 9.4*** | −5.2 ± 5.3*** | 168 | −6.0 ± 8.5*** | −3.9 ± 5.5*** |
| Office BP, mmHg |  |  |  |  |  |  |  |  |  |  |  |  |
| Baseline | 119 | 145.6 ± 18.3 | 83.1 ± 12.0 | 116 | 146.8 ± 16.7 | 86.4 ± 12.8 | 176 | 142.5 ± 15.0 | 83.7 ± 11.4 | 174 | 140.0 ± 13.4 | 81.4 ± 11.3 |
| Week 2 | 118 | 135.0 ± 19.0 | 79.7 ± 11.6 | 116 | 137.5 ± 17.3 | 82.4 ± 12.4 | 176 | 136.6 ± 15.0 | 80.6 ± 11.9 | 174 | 134.7 ± 13.6 | 79.1 ± 11.6 |
| Change from baseline | 118 | −10.7 ± 12.5*** | −3.4 ± 8.0*** | 116 | −9.3 ± 12.8*** | −4.1 ± 8.8*** | 176 | −6.0 ± 11.9*** | −3.1 ± 7.6*** | 174 | −5.3 ± 12.3*** | −2.3 ± 8.0** |
| Week 4 | 119 | 135.1 ± 17.8 | 78.2 ± 11.0 | 116 | 136.2 ± 16.8 | 80.7 ± 12.1 | 176 | 135.5 ± 14.6 | 80.0 ± 11.1 | 174 | 134.1 ± 12.9 | 79.0 ± 10.8 |
| Change from baseline | 119 | −10.5 ± 13.0*** | −4.9 ± 8.3*** | 116 | −10.6 ± 14.1*** | −5.7 ± 9.1*** | 176 | −7.0 ± 12.3*** | −3.8 ± 8.2*** | 174 | −5.9 ± 11.4*** | −2.5 ± 7.7*** |
| Week 8 | 117 | 132.8 ± 18.7 | 77.8 ± 11.7 | 114 | 135.0 ± 17.7 | 79.8 ± 12.6 | 170 | 132.3 ± 15.0 | 78.3 ± 11.4 | 172 | 133.8 ± 13.8 | 78.1 ± 12.2 |
| Change from baseline | 117 | −13.2 ± 13.7*** | −5.3 ± 8.6*** | 114 | −11.5 ± 13.6*** | −6.5 ± 8.7*** | 170 | −9.8 ± 12.1*** | −5.3 ± 7.9*** | 172 | −6.3 ± 11.9*** | −3.3 ± 8.8*** |
| Week 12 | 115 | 131.1 ± 18.6 | 76.1 ± 11.7 | 113 | 133.9 ± 15.7 | 79.8 ± 12.6 | 168 | 130.4 ± 13.9 | 77.7 ± 11.3 | 171 | 132.5 ± 13.9 | 77.7 ± 11.6 |
| Change from baseline | 115 | −14.9 ± 13.8*** | −7.0 ± 8.9*** | 113 | −12.3 ± 13.2*** | −6.5 ± 9.5*** | 168 | −11.8 ± 12.6*** | −5.9 ± 8.8*** | 171 | −7.7 ± 12.3*** | −3.9 ± 8.0*** |
| EOT | 119 | 130.9 ± 18.5 | 76.1 ± 11.6 | 116 | 134.4 ± 15.9 | 80.0 ± 12.5 | 175 | 130.5 ± 13.7 | 77.6 ± 11.3 | 174 | 132.2 ± 14.1 | 77.6 ± 11.8 |
| Change from baseline | 119 | −14.7 ± 13.8*** | −7.0 ± 8.8*** | 116 | −12.4 ± 13.0*** | −6.5 ± 9.3*** | 175 | −11.9 ± 12.6*** | −6.0 ± 8.8*** | 174 | −7.8 ± 12.2*** | −3.9 ± 8.0*** |

Data are mean ± standard deviation.

***p* <0.001, ****p* <0.0001 versus baseline, paired *t*-test.

LS mean change were calculated for morning home BP at Week 12 and EOT.

LS mean change and 95% CIs were calculated using the analysis of covariance model, with morning home SBP/DBP change from baseline as the objective variable; treatment group as the explanatory variable; and baseline BP, baseline antihypertensive medication, and baseline age as covariates.

*ARB* angiotensin receptor blocker, *BP* blood pressure, *CCB* calcium channel blocker, *CI* confidence interval, *DBP* diastolic blood pressure, *EOT* end of treatment, *LS* least squares, *SBP* systolic blood pressure.

**Supplementary Table 3.** Change from baseline in BP (per protocol set)

|  | **ARB** | | | | | | **CCB** | | | | | |
| --- | --- | --- | --- | --- | --- | --- | --- | --- | --- | --- | --- | --- |
|  | **Esaxerenone** | | | **Trichlormethiazide** | | | **Esaxerenone** | | | **Trichlormethiazide** | | |
|  | ***n*** | **SBP, mmHg** | **DBP, mmHg** | ***n*** | **SBP, mmHg** | **DBP, mmHg** | ***n*** | **SBP, mmHg** | **DBP, mmHg** | ***n*** | **SBP, mmHg** | **DBP, mmHg** |
| Morning home BP, mmHg |  |  |  |  |  |  |  |  |  |  |  |  |
| Baseline | 108 | 143.3 ± 15.9 | 87.9 ± 10.0 | 116 | 141.9 ± 14.7 | 88.3 ± 10.5 | 167 | 139.0 ± 14.5 | 86.5 ± 9.6 | 174 | 137.8 ± 11.8 | 85.4 ± 8.4 |
| Week 2 | 108 | 135.1 ± 16.4 | 84.0 ± 9.7 | 116 | 133.1 ± 14.7 | 83.7 ± 10.4 | 167 | 133.4 ± 13.5 | 84.0 ± 9.2 | 174 | 133.5 ± 11.4 | 83.4 ± 8.7 |
| Change from baseline | 108 | −8.2 ± 8.7*** | −3.9 ± 5.0*** | 116 | −8.8 ± 9.7*** | −4.5 ± 5.1*** | 167 | −5.6 ± 6.8*** | −2.6 ± 3.8*** | 174 | −4.3 ± 7.1*** | −2.0 ± 4.1*** |
| Week 4 | 108 | 132.1 ± 16.6 | 82.1 ± 9.6 | 116 | 131.8 ± 14.7 | 82.9 ± 10.0 | 167 | 131.9 ± 13.8 | 83.0 ± 9.5 | 174 | 132.9 ± 12.1 | 82.9 ± 8.8 |
| Change from baseline | 108 | −11.2 ± 10.2*** | −5.9 ± 5.5*** | 116 | −10.1 ± 9.5*** | −5.4 ± 5.0*** | 167 | −7.1 ± 7.4*** | −3.6 ± 4.2*** | 174 | −4.9 ± 7.6*** | −2.5 ± 4.0*** |
| Week 6 | 107 | 130.5 ± 16.0 | 81.0 ± 9.8 | 115 | 130.6 ± 14.3 | 81.7 ± 9.9 | 165 | 131.1 ± 14.4 | 82.3 ± 9.2 | 174 | 132.2 ± 12.1 | 82.3 ± 8.7 |
| Change from baseline | 107 | −13.0 ± 10.1*** | −7.0 ± 5.7*** | 115 | −11.3 ± 9.7*** | −6.5 ± 5.6*** | 165 | −7.8 ± 7.3*** | −4.1 ± 4.2*** | 174 | −5.6 ± 8.3*** | −3.1 ± 4.3*** |
| Week 8 | 106 | 130.6 ± 15.4 | 81.1 ± 9.8 | 115 | 130.1 ± 14.8 | 81.7 ± 10.4 | 164 | 130.7 ± 14.1 | 82.3 ± 9.4 | 173 | 131.2 ± 11.6 | 81.4 ± 8.8 |
| Change from baseline | 106 | −13.0 ± 10.1*** | −6.9 ± 6.1*** | 115 | −11.8 ± 11.0*** | −6.5 ± 5.7*** | 164 | −8.0 ± 7.6*** | −4.2 ± 4.4*** | 173 | −6.7 ± 8.4*** | −4.1 ± 4.5*** |
| Week 10 | 104 | 127.8 ± 15.1 | 79.7 ± 9.6 | 113 | 129.3 ± 14.3 | 81.3 ± 10.6 | 163 | 129.0 ± 13.0 | 81.2 ± 9.4 | 172 | 130.9 ± 11.1 | 81.4 ± 8.9 |
| Change from baseline | 104 | −15.7 ± 11.4*** | −8.4 ± 5.9*** | 113 | −12.3 ± 9.6*** | −6.8 ± 5.0*** | 163 | −9.5 ± 8.8*** | −5.2 ± 5.0*** | 172 | −7.0 ± 8.6*** | −4.0 ± 4.6*** |
| Week 12 | 104 | 128.5 ± 14.7 | 79.8 ± 10.0 | 113 | 129.2 ± 14.0 | 80.9 ± 10.9 | 160 | 128.1 ± 12.0 | 81.1 ± 9.3 | 172 | 130.6 ± 10.7 | 81.1 ± 8.6 |
| Change from baseline | 104 | −15.1 ± 11.7*** | −8.2 ± 6.4*** | 113 | −12.4 ± 10.4*** | −7.2 ± 6.4*** | 160 | −10.5 ± 8.9*** | −5.4 ± 4.8*** | 172 | −7.4 ± 7.5*** | −4.4 ± 4.1*** |
| LS mean change from baseline [95% CI] |  | −14.5  [−16.5, −12.6] | −7.9  [−9.1, −6.8] |  | −12.9  [−14.7, −11.1] | −7.4  [−8.5, −6.4] |  | −10.5  [−11.6, −9.4] | −5.4  [−6.1, −4.8] |  | −7.4  [−8.5, −6.3] | −4.4  [−5.0, −3.8] |
| Difference in LS mean change from baseline [95% CI] |  | −1.6  [−4.3, 1.0] | −0.5  [−2.1, 1.1] |  | - | - |  | −3.1  [−4.6, −1.6] | −1.0  [−1.9, −0.1] |  | - | - |
| EOT | 108 | 128.1 ± 14.7 | 79.7 ± 9.9 | 116 | 129.2 ± 13.9 | 80.9 ± 10.7 | 166 | 128.6 ± 12.3 | 81.2 ± 9.2 | 174 | 130.4 ± 10.9 | 81.0 ± 8.6 |
| Change from baseline | 108 | −15.2 ± 11.8*** | −8.2 ± 6.3*** | 116 | −12.7 ± 10.6*** | −7.3 ± 6.4*** | 166 | −10.4 ± 9.2*** | −5.2 ± 5.0*** | 174 | −7.4 ± 7.5*** | −4.4 ± 4.1*** |
| LS mean change from baseline [95% CI] |  | −14.7  [−16.6, −12.8] | −8.0  [−9.1, −6.9] |  | −13.1  [−14.9, −11.3] | −7.6  [−8.6, −6.5] |  | −10.3  [−11.4, −9.2] | −5.2  [−5.9, −4.6] |  | −7.5  [−8.6, −6.5] | −4.4  [−5.1, −3.8] |
| Difference in LS mean change from baseline [95% CI] |  | −1.6  [−4.2, 1.0] | −0.4  [−2.0, 1.1] |  | - | - |  | −2.8  [−4.3, −1.3] | −0.8  [−1.7, 0.1] |  | - | - |
| Bedtime home BP, mmHg |  |  |  |  |  |  |  |  |  |  |  |  |
| Baseline | 102 | 136.3 ± 17.3 | 81.6 ± 10.8 | 114 | 136.8 ± 16.4 | 82.1 ± 11.9 | 159 | 134.7 ± 15.2 | 81.9 ± 10.5 | 168 | 132.8 ± 11.9 | 80.7 ± 10.0 |
| Week 2 | 107 | 128.9 ± 18.1 | 78.4 ± 11.3 | 116 | 128.1 ± 15.1 | 77.8 ± 11.2 | 162 | 129.6 ± 13.8 | 80.0 ± 9.6 | 172 | 129.6 ± 11.7 | 78.9 ± 9.5 |
| Change from baseline | 102 | −8.1 ± 9.2*** | −3.3 ± 4.9*** | 114 | −8.6 ± 8.4*** | −4.3 ± 4.7*** | 159 | −5.1 ± 7.1*** | −1.9 ± 4.1*** | 168 | −3.5 ± 7.0*** | −1.8 ± 4.7*** |
| Week 4 | 107 | 125.9 ± 17.7 | 76.0 ± 10.6 | 116 | 126.4 ± 14.7 | 76.8 ± 10.6 | 165 | 128.0 ± 13.7 | 78.7 ± 10.1 | 172 | 129.0 ± 11.7 | 78.4 ± 9.3 |
| Change from baseline | 102 | −11.3 ± 10.1*** | −5.8 ± 5.3*** | 114 | −10.2 ± 9.2*** | −5.3 ± 5.6*** | 159 | −6.8 ± 7.9*** | −3.3 ± 5.0*** | 168 | −4.1 ± 7.8*** | −2.3 ± 5.3*** |
| Week 6 | 106 | 125.5 ± 17.3 | 75.9 ± 11.2 | 115 | 125.0 ± 14.9 | 75.9 ± 10.7 | 163 | 126.5 ± 13.3 | 77.9 ± 9.5 | 172 | 127.9 ± 11.0 | 77.7 ± 9.2 |
| Change from baseline | 101 | −11.6 ± 10.9*** | −5.8 ± 6.1*** | 113 | −11.8 ± 9.9*** | −6.2 ± 6.3*** | 157 | −8.2 ± 8.5*** | −4.2 ± 5.0*** | 168 | −5.1 ± 7.6*** | −2.9 ± 5.3*** |
| Week 8 | 105 | 124.6 ± 16.8 | 75.0 ± 10.8 | 115 | 124.5 ± 14.6 | 75.6 ± 10.8 | 162 | 125.9 ± 13.2 | 77.4 ± 9.9 | 171 | 127.5 ± 11.6 | 77.2 ± 9.3 |
| Change from baseline | 100 | −12.5 ± 10.0*** | −6.8 ± 6.0*** | 113 | −12.2 ± 11.0*** | −6.5 ± 6.6*** | 156 | −8.5 ± 8.5*** | −4.5 ± 5.1*** | 167 | −5.6 ± 8.0*** | −3.5 ± 5.0*** |
| Week 10 | 103 | 122.4 ± 15.9 | 74.1 ± 10.0 | 113 | 122.9 ± 15.5 | 74.5 ± 11.0 | 161 | 125.2 ± 11.4 | 77.2 ± 9.4 | 170 | 127.6 ± 11.5 | 76.7 ± 9.7 |
| Change from baseline | 98 | −15.1 ± 10.8*** | −7.9 ± 6.1*** | 111 | −13.4 ± 11.1*** | −7.4 ± 6.5*** | 155 | −9.2 ± 9.4*** | −4.9 ± 5.4*** | 166 | −5.7 ± 8.5*** | −4.0 ± 5.6*** |
| Week 12 | 103 | 123.0 ± 15.4 | 73.8 ± 10.6 | 113 | 123.6 ± 15.8 | 74.6 ± 11.3 | 158 | 124.8 ± 12.0 | 76.8 ± 9.5 | 170 | 127.2 ± 11.5 | 76.8 ± 9.0 |
| Change from baseline | 98 | −14.6 ± 11.2*** | −8.2 ± 6.4*** | 111 | −12.9 ± 10.9*** | −7.3 ± 7.0*** | 152 | −9.8 ± 9.5*** | −5.3 ± 5.3*** | 166 | −6.0 ± 8.5*** | −3.9 ± 5.6*** |
| EOT | 107 | 122.6 ± 15.4 | 73.6 ± 10.5 | 116 | 123.6 ± 15.6 | 74.7 ± 11.2 | 164 | 125.1 ± 12.2 | 76.9 ± 9.3 | 172 | 127.0 ± 11.6 | 76.8 ± 9.0 |
| Change from baseline | 102 | −14.3 ± 11.3*** | −8.1 ± 6.4*** | 114 | −13.2 ± 10.9*** | −7.4 ± 7.0*** | 158 | −9.7 ± 9.5*** | −5.1 ± 5.3*** | 168 | −6.0 ± 8.5*** | −3.9 ± 5.5*** |
| Office BP, mmHg |  |  |  |  |  |  |  |  |  |  |  |  |
| Baseline | 108 | 145.8 ± 18.6 | 82.9 ± 12.0 | 116 | 146.8 ± 16.7 | 86.4 ± 12.8 | 167 | 142.1 ± 15.0 | 83.8 ± 11.5 | 174 | 140.0 ± 13.4 | 81.4 ± 11.3 |
| Week 2 | 107 | 135.0 ± 19.4 | 79.5 ± 11.8 | 116 | 137.5 ± 17.3 | 82.4 ± 12.4 | 167 | 136.6 ± 15.2 | 80.8 ± 12.0 | 174 | 134.7 ± 13.6 | 79.1 ± 11.6 |
| Change from baseline | 107 | −10.8 ± 12.5 | −3.4 ± 8.2*** | 116 | −9.3 ± 12.8*** | −4.1 ± 8.8*** | 167 | −5.6 ± 11.3*** | −3.0 ± 7.7*** | 174 | −5.3 ± 12.3*** | −2.3 ± 8.0** |
| Week 4 | 108 | 135.3 ± 18.4 | 78.3 ± 11.2 | 116 | 136.2 ± 16.8 | 80.7 ± 12.1 | 167 | 135.6 ± 14.9 | 80.0 ± 11.3 | 174 | 134.1 ± 12.9 | 79.0 ± 10.8 |
| Change from baseline | 108 | −10.5 ± 12.7*** | −4.6 ± 8.1*** | 116 | −10.6 ± 14.1*** | −5.7 ± 9.1*** | 167 | −6.6 ± 12.0*** | −3.8 ± 8.4*** | 174 | −5.9 ± 11.4*** | −2.5 ± 7.7*** |
| Week 8 | 106 | 133.1 ± 19.3 | 77.5 ± 11.9 | 114 | 135.0 ± 17.7 | 79.8 ± 12.6 | 162 | 132.2 ± 15.1 | 78.4 ± 11.6 | 172 | 133.8 ± 13.8 | 78.1 ± 12.2 |
| Change from baseline | 106 | −13.0 ± 13.5*** | −5.3 ± 8.7*** | 114 | −11.5 ± 13.6*** | −6.5 ± 8.7*** | 162 | −9.7 ± 12.1*** | −5.4 ± 8.0*** | 172 | −6.3 ± 11.9*** | −3.3 ± 8.8*** |
| Week 12 | 104 | 131.4 ± 19.3 | 75.9 ± 11.9 | 113 | 133.9 ± 15.7 | 79.8 ± 12.6 | 160 | 130.4 ± 14.1 | 77.8 ± 11.5 | 171 | 132.5 ± 13.9 | 77.7 ± 11.6 |
| Change from baseline | 104 | −14.8 ± 13.8*** | −7.0 ± 8.9*** | 113 | −12.3 ± 13.2*** | −6.5 ± 9.5*** | 160 | −11.5 ± 12.4*** | −5.9 ± 9.0*** | 171 | −7.7 ± 12.3*** | −3.9 ± 8.0*** |
| EOT | 108 | 131.2 ± 19.1 | 76.0 ± 11.8 | 116 | 134.4 ± 15.9 | 80.0 ± 12.5 | 166 | 130.5 ± 14.0 | 77.7 ± 11.4 | 174 | 132.2 ± 14.1 | 77.6 ± 11.8 |
| Change from baseline | 108 | −14.6 ± 13.8*** | −7.0 ± 8.8*** | 116 | −12.4 ± 13.0*** | −6.5 ± 9.3*** | 166 | −11.5 ± 12.5*** | −5.9 ± 9.0*** | 174 | −7.8 ± 12.2*** | −3.9 ± 8.0*** |

Data are mean ± standard deviation.

***p* <0.001, ****p* <0.0001 versus baseline, paired *t*-test.

LS mean change were calculated for morning home BP at Week 12 and EOT.

LS mean change and 95% CIs were calculated using the analysis of covariance model, with morning home SBP/DBP change from baseline as the objective variable; treatment group as the explanatory variable; and baseline BP, baseline antihypertensive medication, and baseline age as covariates.

*ARB* angiotensin receptor blocker, *BP* blood pressure, *CCB* calcium channel blocker, *CI* confidence interval, *DBP* diastolic blood pressure, *EOT* end of treatment, *LS* least squares, *SBP* systolic blood pressure.

**Supplementary Table 4.** Change in UACR and NT-proBNP from baseline to Week 12 (full analysis set)

|  | **ARB** | | | | **CCB** | | | |
| --- | --- | --- | --- | --- | --- | --- | --- | --- |
|  | **Esaxerenone** | | **Trichlormethiazide** | | **Esaxerenone** | | **Trichlormethiazide** | |
| **UACR, mg/gCr** | ***n*** |  | ***n*** |  | ***n*** |  | ***n*** |  |
| Baseline | 119 | 159.71 ± 700.75 | 116 | 143.92 ± 641.37 | 176 | 86.89 ± 239.90 | 174 | 72.97 ± 159.47 |
| Week 4 | 119 | 119.26 ± 632.85 | 116 | 103.45 ± 498.87 | 176 | 49.86 ± 106.37 | 174 | 47.98 ± 108.63 |
| Change from baseline | 119 | −40.45 ± 169.58 | 116 | −40.48 ± 260.87 | 176 | −37.03 ± 187.69 | 174 | −25.00 ± 124.42 |
| Percentage change in geometric mean from baseline [95% CI] |  | −35.4  [−43.4, −26.1]*** |  | −31.3  [−40.3, −20.8]*** |  | −31.4  [−38.5, −23.4]*** |  | −27.3  [−35.1, −18.7]*** |
| Week 8 | 117 | 58.83 ± 204.54 | 114 | 78.41 ± 373.95 | 170 | 44.01 ± 117.85 | 172 | 40.33 ± 105.84 |
| Change from baseline | 117 | −46.99 ± 199.82 | 114 | −67.23 ± 293.74 | 170 | −26.69 ± 96.08 | 172 | −33.38 ± 141.90 |
| Percentage change in geometric mean from baseline [95% CI] |  | −34.6  [−43.2, −24.7]*** |  | −34.2  [−43.3, −23.7]*** |  | −40.8  [−47.8, −32.8]*** |  | −36.6  [−43.2, −29.4]*** |
| Week 12 | 115 | 88.81 ± 352.53 | 113 | 85.16 ± 398.36 | 168 | 28.13 ± 58.81 | 171 | 34.01 ± 81.82 |
| Change from baseline | 115 | −17.94 ± 243.13 | 113 | −61.36 ± 310.80 | 168 | −36.86 ± 110.74 | 171 | −39.92 ± 134.67 |
| Percentage change in geometric mean from baseline [95% CI] |  | −33.2  [−44.0, −20.5]*** |  | −36.3  [−45.6, −25.5]*** |  | −42.5  [−49.8, −34.2]*** |  | −45.3  [−51.0, −39.0]*** |
| **NT-proBNP, pg/mL** |  |  |  |  |  |  |  |  |
| Baseline | 96 | 161.54 ± 467.55 | 100 | 104.31 ± 207.40 | 141 | 74.06 ± 95.38 | 153 | 70.47 ± 86.83 |
| Week 12 | 87 | 99.63 ± 177.39 | 95 | 95.41 ± 173.21 | 133 | 63.04 ± 55.52 | 146 | 62.45 ± 62.92 |
| Change from baseline | 87 | −65.86 ± 376.78 | 93 | −9.95 ± 129.89 | 133 | −11.66 ± 70.29 | 145 | −9.25 ± 54.33* |

Data are geometric mean ± standard deviation.

**p* <0.05 versus baseline, paired *t*-test.

****p* <0.001 versus baseline, paired *t*-test.

For UACR, *p-*values are only presented for percentage change from baseline.

*ARB* angiotensin receptor blocker, *CCB* calcium channel blocker, *CI* confidence interval, *NT-proBNP* N-terminal pro-brain natriuretic peptide, *UACR* urinary albumin-to-creatinine ratio.

**Supplementary Table 5.** Change in UACR and NT-proBNP from baseline to Week 12 (per protocol set)

|  | **ARB** | | | | **CCB** | | | |
| --- | --- | --- | --- | --- | --- | --- | --- | --- |
|  | **Esaxerenone** | | **Trichlormethiazide** | | **Esaxerenone** | | **Trichlormethiazide** | |
| **UACR, mg/gCr** | ***n*** |  | ***n*** |  | ***n*** |  | ***n*** |  |
| Baseline | 108 | 158.80 ± 725.35 | 116 | 143.92 ± 641.37 | 167 | 90.57 ± 245.77 | 174 | 72.97 ± 159.47 |
| Week 4 | 108 | 123.56 ± 662.69 | 116 | 103.45 ± 498.87 | 167 | 51.87 ± 108.84 | 174 | 47.98 ± 108.63 |
| Change from baseline | 108 | −35.23 ± 159.16 | 116 | −40.48 ± 260.87 | 167 | −38.70 ± 192.57 | 174 | −25.00 ± 124.42 |
| Percentage change in geometric mean from baseline [95% CI] |  | −34.1  [−42.9, −24.0]*** |  | −31.3  [−40.3, −20.8]*** |  | −31.1  [−38.6, −22.7]*** |  | −27.3  [−35.1, −18.7]*** |
| Week 8 | 106 | 60.09 ± 213.11 | 114 | 78.41 ± 373.95 | 162 | 45.43 ± 120.56 | 172 | 40.33 ± 105.84 |
| Change from baseline | 106 | −39.21 ± 183.78 | 114 | −67.23 ± 293.74 | 162 | −27.85 ± 98.28 | 172 | −33.38 ± 141.90 |
| Percentage change in geometric mean from baseline [95% CI] |  | −32.2  [−41.5, −21.3]*** |  | −34.2  [−43.3, −23.7]*** |  | −41.6  [−48.8, −33.4]*** |  | −36.6  [−43.2, −29.4]*** |
| Week 12 | 104 | 90.44 ± 367.34 | 113 | 85.16 ± 398.36 | 160 | 28.97 ± 60.13 | 171 | 34.01 ± 81.82 |
| Change from baseline | 104 | −9.76 ± 242.76 | 113 | −61.36 ± 310.80 | 160 | −38.34 ± 113.28 | 171 | −39.92 ± 134.67 |
| Percentage change in geometric mean from baseline [95% CI] |  | −31.5  [−43.3, −17.1]*** |  | −36.3  [−45.6, −25.5]*** |  | −42.6  [−50.1, −33.9]*** |  | −45.3  [−51.0, −39.0]*** |
| **NT-proBNP, pg/mL** |  |  |  |  |  |  |  |  |
| Baseline | 89 | 168.46 ± 484.91 | 100 | 104.31 ± 207.40 | 134 | 72.45 ± 95.91 | 153 | 70.47 ± 86.83 |
| Week 12 | 81 | 104.91 ± 182.73 | 95 | 95.41 ± 173.21 | 126 | 60.22 ± 51.82 | 146 | 62.45 ± 62.92 |
| Change from baseline | 81 | −68.43 ± 390.43 | 93 | −9.95 ± 129.89 | 126 | −12.80 ± 71.99* | 145 | −9.25 ± 54.33* |

Data are geometric mean ± standard deviation.

**p* <0.05 versus baseline, paired *t*-test.

****p* <0.001 versus baseline, paired *t*-test.

For UACR, *p-*values are only presented for percentage change from baseline.

*ARB* angiotensin receptor blocker, *CCB* calcium channel blocker, *CI* confidence interval, *NT-proBNP* N-terminal pro-brain natriuretic peptide, *UACR* urinary albumin-to-creatinine ratio.

**Supplementary Table 6.** Change in serum potassium and eGFR_creat_ from baseline to Week 12 (safety analysis set)

|  | **ARB** | | | | **CCB** | | | |
| --- | --- | --- | --- | --- | --- | --- | --- | --- |
|  | **Esaxerenone** | | **Trichlormethiazide** | | **Esaxerenone** | | **Trichlormethiazide** | |
|  | ***n*** | **Mean ± SD** | ***n*** | **Mean ± SD** | ***n*** | **Mean ± SD** | ***n*** | **Mean ± SD** |
| **Serum potassium, mEq/L** |  |  |  |  |  |  |  |  |
| Baseline | 116 | 4.27 ± 0.35 | 113 | 4.27 ± 0.34 | 177 | 4.17 ± 0.34 | 172 | 4.16 ± 0.32 |
| Week 2 | 114 | 4.41 ± 0.39 | 113 | 4.16 ± 0.38 | 171 | 4.32 ± 0.31 | 169 | 4.04 ± 0.37 |
| Change from baseline | 113 | 0.14 ± 0.37 | 112 | −0.12 ± 0.35 | 171 | 0.15 ± 0.34 | 169 | −0.13 ± 0.29 |
| Week 4 | 115 | 4.27 ± 0.38 | 109 | 4.18 ± 0.34 | 171 | 4.29 ± 0.37 | 170 | 4.04 ± 0.35 |
| Change from baseline | 113 | −0.01 ± 0.33 | 109 | −0.10 ± 0.32 | 171 | 0.12 ± 0.38 | 169 | −0.12 ± 0.36 |
| Week 8 | 112 | 4.32 ± 0.41 | 108 | 4.13 ± 0.34 | 167 | 4.28 ± 0.34 | 168 | 4.00 ± 0.35 |
| Change from baseline | 111 | 0.07 ± 0.40 | 108 | −0.16 ± 0.35 | 167 | 0.11 ± 0.37 | 167 | −0.16 ± 0.34 |
| Week 12 | 110 | 4.22 ± 0.37 | 107 | 4.11 ± 0.34 | 165 | 4.25 ± 0.33 | 165 | 3.92 ± 0.34 |
| Change from baseline | 109 | −0.04 ± 0.36 | 107 | −0.17 ± 0.36 | 165 | 0.08 ± 0.34 | 165 | −0.24 ± 0.34 |
| **eGFR_creat_, mL/min/1.73 m^2^** |  |  |  |  |  |  |  |  |
| Baseline | 122 | 70.68 ± 16.17 | 118 | 68.37 ± 16.88 | 180 | 72.03 ± 15.47 | 177 | 74.34 ± 16.90 |
| Week 2 | 119 | 65.31 ± 14.53 | 118 | 66.54 ± 16.45 | 176 | 66.94 ± 14.93 | 177 | 70.29 ± 15.41 |
| Change from baseline | 119 | −5.34 ± 9.00 | 117 | −1.78 ± 7.81 | 176 | −5.12 ± 7.40 | 175 | −4.21 ± 9.36 |
| Week 4 | 119 | 65.52 ± 15.38 | 115 | 65.59 ± 15.64 | 176 | 67.02 ± 14.89 | 176 | 70.04 ± 15.63 |
| Change from baseline | 119 | −5.15 ± 9.11 | 114 | −2.66 ± 7.53 | 176 | −5.04 ± 7.59 | 174 | −4.54 ± 9.49 |
| Week 8 | 117 | 65.08 ± 16.25 | 114 | 64.64 ± 16.38 | 170 | 65.46 ± 15.05 | 174 | 69.74 ± 15.63 |
| Change from baseline | 117 | −5.72 ± 9.05 | 113 | −3.78 ± 7.35 | 170 | −6.48 ± 8.61 | 172 | −4.94 ± 10.89 |
| Week 12 | 115 | 63.72 ± 14.62 | 113 | 63.96 ± 15.24 | 168 | 64.93 ± 14.89 | 171 | 70.03 ± 15.01 |
| Change from baseline | 115 | −7.06 ± 8.31 | 112 | −4.74 ± 9.02 | 168 | −7.10 ± 8.84 | 169 | −4.63 ± 10.37 |

*p*-values were not calculated.

*ARB* angiotensin receptor blocker, *CCB* calcium channel blocker, *eGFR_creat_* creatinine-based estimated glomerular filtration rate, *SD* standard deviation.

**Supplementary Table 7.** Incidence of serum potassium level <3.5, ≥5.5, and ≥6.0 mEq/L (safety analysis set)

|  | **ARB** | | **CCB** | |
| --- | --- | --- | --- | --- |
|  | **Esaxerenone**  ***n* = 118** | **Trichlormethiazide**  ***n* = 114** | **Esaxerenone**  ***n* = 177** | **Trichlormethiazide**  ***n* = 173** |
| Serum potassium <3.5 mEq/L | 4 (3.4) [0.9, 8.5] | 9 (7.9) [3.7, 14.5] | 5 (2.8) [0.9, 6.5] | 24 (13.9) [9.1, 19.9] |
| Serum potassium ≥5.5 mEq/L | 5 (4.2) [1.4, 9.6] | 0 (0.0) [0.0, 3.2] | 1 (0.6) [0.0, 3.1] | 2 (1.2) [0.1, 4.1] |
| Serum potassium ≥6.0 mEq/L | 0 (0.0) [0.0, 3.1] | 0 (0.0) [0.0, 3.2] | 0 (0.0) [0.0, 2.1] | 0 (0.0) [0.0, 2.1] |

Data are n (%) [95% CI].

*ARB* angiotensin receptor blocker, *CCB* calcium channel blocker, *CI* confidence interval.

**Supplementary Table 8.** Incidence of UA level ≥7.0 mg/dL (safety analysis set)

|  | **ARB** | | **CCB** | |
| --- | --- | --- | --- | --- |
|  | **Esaxerenone**  ***n* = 122** | **Trichlormethiazide**  ***n* = 119** | **Esaxerenone**  ***n* = 180** | **Trichlormethiazide**  ***n* = 179** |
| UA ≥7.0 mg/dL | 34 (27.9) [20.1, 36.7] | 47 (39.5) [30.7, 48.9] | 49 (27.2) [20.9, 34.3] | 56 (31.3) [24.6, 38.6] |

Data are n (%) [95% CI].

*ARB* angiotensin receptor blocker, *CCB* calcium channel blocker, *CI* confidence interval, *UA* uric acid.
